# A novel *in vitro* potency assay of antisera against Thai *Naja kaouthia* based on nicotinic acetylcholine receptor binding

**DOI:** 10.1038/s41598-017-08962-3

**Published:** 2017-08-17

**Authors:** Kavi Ratanabanangkoon, Pavinee Simsiriwong, Kritsada Pruksaphon, Kae Yi Tan, Sukanya Eursakun, Choo Hock Tan, Bunkuea Chantrathonkul, Wongsakorn Wongwadhunyoo, Sirida Youngchim, Nget Hong Tan

**Affiliations:** 1 Laboratory of Immunology, Chulabhorn Research Institute, Bangkok, Thailand; 2 Chulabhorn Graduate Institute, Bangkok, 10210 Thailand; 3 Department of Microbiology, Faculty of Science, Mahidol University, Rama 6 Road, Bangkok, 10400 Thailand; 4 Department of Microbiology, Faculty of Medicine, Chiang Mai University, Chiang Mai, Thailand; 5 Department of Molecular Medicine, Faculty of Medicine, University of Malaya, Kuala Lumpur, 50603 Malaysia; 6 Department of Pharmacology, Faculty of Medicine, University of Malaya, Kuala Lumpur, 50603 Malaysia; 7 Faculty of Veterinary Science, Mahidol University, Salaya, NakornPrathom 73170 Thailand

## Abstract

Snake envenomation is an important medical problem. One of the hurdles in antivenom development is the *in vivo* assay of antivenom potency which is expensive, gives variable results and kills many animals. We report a novel *in vitro* assay involving the specific binding of the postsynaptic neurotoxins (PSNTs) of elapid snakes with purified *Torpedo californica* nicotinic acetylcholine receptor (nAChR). The potency of an antivenom is determined by its antibody ability to bind and neutralize the PSNT, thus preventing it from binding to nAChR. The PSNT of *Naja kaouthia* (NK3) was immobilized on microtiter wells and nAChR was added to bind with it. The *in vitro* IC_50_ of *N. kaouthia* venom that inhibited 50% of nAChR binding to the immobilized NK3 was determined. Varying concentrations of antisera against *N. kaouthia* were separately pre-incubated with 5xIC_50_ of *N. kaouthia* venom. The remaining free NK3 were incubated with nAChR before adding to the NK3 coated plates. The *in vitro* and *in vivo* median effective ratio, ER_50_s of 12 batches of antisera showed correlation (*R* ^2^) of 0.9809 (*p* < 0.0001). This *in vitro* assay should be applicable to antisera against other elapid venoms and should reduce the use of live animals and accelerate development of life-saving antivenoms.

## Introduction

Snake envenomation is an important medical problem especially in the developing world. It has been estimated that around 421,000–2.5 million people are envenomed annually with about 20,000–94,000 fatalities^1^. Antivenoms (AVs) are the rationale and the most effective therapy of snake envenomation. However, this serious public health problem has so far been neglected and effective, affordable antivenoms remain unavailable in many parts of the developing world. Recently, efforts from a number of research institutions are underway to solve this problem^2^.

In the development and production of an AV, at least two major steps are involved: an effective immunization program and the pre-clinical testing to assess the neutralizing potential of the AV against the lethal effects of homologous and heterologous venoms. The accepted AV potency assay is the standard murine lethality assay to determine the median lethal dose (LD_50_) that estimates the lethality of the venom and the median effective dose (ED_50_) of the AV^3, 4^. For this *in vivo* assay, three to five mice per venom dose are used and the total of about 6 different doses are tested. Thus, about 30 mice are needed for the determination of the LD_50_ of a venom. Similarly, about 30 mice are needed for the ED_50_ determination of an AV against a venom. Therefore, a large number of mice will be used for the *in vivo* neutralization assays. For example, the number of mice required by European Pharmacopoeia using this method to test the activity of one European viper venom antiserum (LD_50_ and ED_50_ tests combined) against five venoms, is 374 mice per batch of antivenom^5^. Using this figure, testing a pan-specific AV against 27 different venoms^6^ would require about 2,020 mice. The assay is very costly, laborious and can give highly variable results. Moreover, some lethality studies have been shown to be inconsistent, suggesting that rodent death may not measure relevant efficacy outcomes in humans^7^. Lastly, witnessing the suffering and death of a large number of animals is the most difficult part of the experiment for many. In Buddhist countries like Thailand, most, if not all, laboratory personnel and graduate students refuse to do such experiments. Thus, it is becoming increasingly difficult to perform the *in vivo* assay for ethical and religious, as well as regulatory reasons.

For the above reasons, various types of *in vitro* neutralization assays have been developed to be used in place of, or to reduce the number of the *in vivo* assay. It has been reported that venom toxicity and effectiveness of AV can be studied using the chick biventer cervicis preparation^8–10^. This assay was used for screening AVs against the neurotoxic effects of venoms^11^. However, this *in vitro* assay requires the preparation of chick biventer cervicis muscles and the assay is laborious and time-consuming. *In vitro* neutralization of some venom enzymatic activities have been studied for use in AV potency assay^12^. It was shown that the neutralization of phospholipase A_2_ activities by antivenom against *Micrurus nigrocinctus* highly correlated with the *in vivo* neutralization activity. Also, the inhibition of indirect hemolytic activity induced by phospholipase A_2_ was also shown to correlate well with the *in vivo* potency of a polyspecific antivenom^13^. However, these assays are applicable only to antivenoms directed against venoms with enzymatic activities that parallel the lethality of the venoms.

Numerous investigators have studied and reported the use of ELISA for AV potency determinations together with the correlation between the results of the *in vitro* and *in vivo* neutralization assays^14–20^. However, the “neutralizing potency” described as “*in vitro* ELISA titer” occasionally did not correlate well with the *in vivo* neutralizing activity. For example, Ibrahim and Farid^21^ studied the lethality-neutralizing potency, ELISA antibody level and the avidity indexes of a polyvalent antivenom against seven snake venoms. They showed poor correlation between the *in vivo* and *in vitro* assays with the *in vitro* assays always giving high values. ELISA has at times been criticized on the grounds that the antigen-antibody *‘binding’* reaction measured cannot be assumed to be the same as the *‘neutralization’* reaction of the antigen.

Elapid snakes (cobras, kraits and mambas) produce the lethal postsynaptic neurotoxins (PSNTs) which bind specifically and quasi-irreversibly with nicotinic acetylcholine receptor (nAChR) at the muscle end-plate^22, 23^. This binding results in the inhibition of neuromuscular transmission which can lead to respiratory arrest and death^23^. Thus, it should be possible to develop an *in vitro* functional assay to test antivenom against elapid venoms based on the ability of the antivenom antibodies to inhibit the binding of PSNTs to nAChR. Such an *in vitro* assay would closely mimic the lethality reactions of PSNTs of the elapids *in vivo*, in particular cobras (*Naja* sp.). Cobras are in general listed as Category 1 of medically important snakes throughout most parts of Asia and Africa^4, 24^.

An *in vitro* potency assay based on PSNT binding to nAChR was previously studied for the venom of coral snake *Micrurus nigrocinctus* ^12^. It was found that, the ED_50_ of the horse antisera against *M. nigrocinctus* in neutralizing the lethal effect of the venom did not correlate with the antivenom ability to inhibit the nAChR-binding activity but correlated well with the inhibition of the venom phospholipase A_2_ activity.

By using a different reaction scheme and conditions from that studied above^12^, we report here the development of a novel *in vitro* potency assay of monospecific antisera against the venom of the Thai monocled cobra *Naja kaouthia* based on nAChR binding. The assays gave excellent correlation (*R* ^2^ = 0.9809; *p* < 0.0001) with the corresponding *in vivo* assay using mice. This *in vitro* assay should be useful in reducing or partially replacing the *in vivo* assays used to test antivenoms against *N. kaouthia* and other elapid venoms.

## Results

### Development of the *in vitro* neutralization assay using nAChR-PSNT binding

#### The optimal concentrations of NK3, nAChR, rat anti-nAChR antibody and goat-anti-rat HRP conjugate in the *in vitro* potency assay

**Figure 1:**
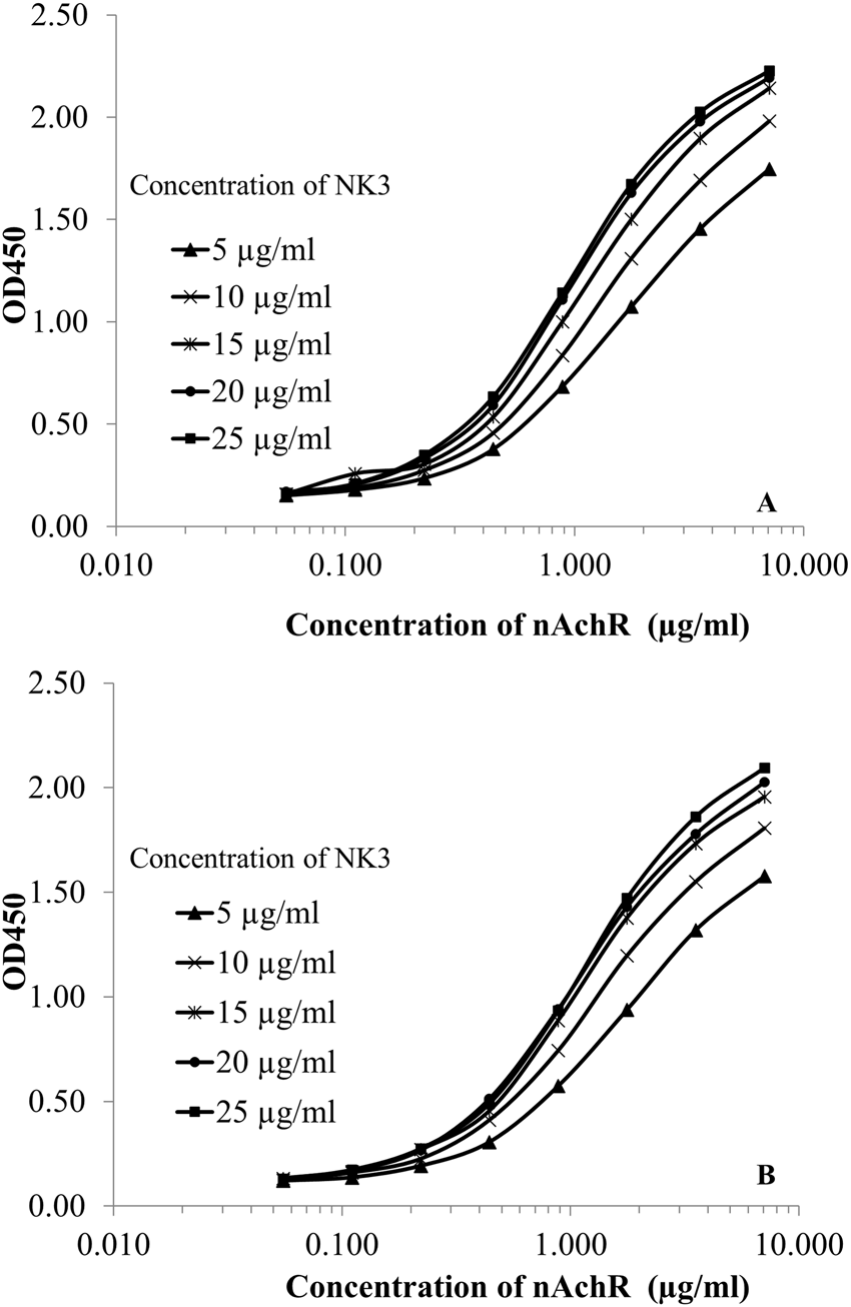
Determinations of optimal concentrations of NK3, purified nAChR, rat anti-nAChR serum and goat anti-rat IgG conjugated HRP. A: goat anti-rat IgG-HRP conjugate at 1:4500, B: goat anti-rat IgG-HRP conjugate at 1:6000.

The optimal concentrations of NK3, nAChR, rat anti-nAChR antibody and goat-anti-rat HRP conjugate used in the *in vitro* potency assay were studied as described in Materials and Methods (step: pre-incubation 2). The results are shown in Fig. 1. As the concentration of NK3 used to coat the microtiter plate increased, the signal as measured by the OD_450nm_ increased. This was also observed when the concentration of nAchR used to bind the immobilized NK3 was increased. To economize on the nAChR available while obtaining reasonably high OD_450nm_ signal, it was decided to use 15 µg/ml of NK3 for coating the plates and 0.707 µg/ml of nAChR for binding to the NK3 coated plate. A 1:1600 dilution of rat anti-nAchR serum and a 1:4500 dilution of goat anti-rat-IgG conjugated HRP were used.

#### Inhibition of nAChR binding to NK3-coated plate by *N. kaouthia* venom or *N. kaouthia* cytotoxin

**Figure 2:**
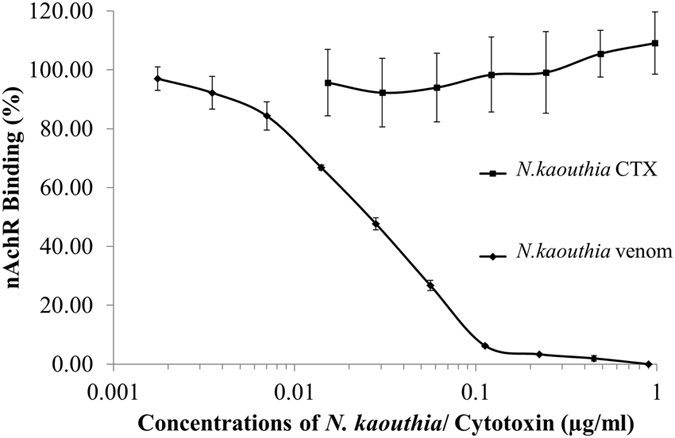
The inhibition of nAChR binding to the NK3 coated-plate by *N. kaouthia* venom and by *N. kaouthia* cytotoxin I. nAChR binding were expressed as mean ± S.D. of 4 determinations.

Crude *N. kaout﻿hia* venom was used to determine the 50% inhibition of nAChR binding (*in vitro* IC_50_). In the first step, crude *N. kaouthia* venom at various concentrations was incubated with the purified nAChR (0.707 µg/ml) for 1 hr at 25 °C. The solution was then transferred to NK3 coated plates. The concentration of the crude venom that reduced nAchR binding by 50% was defined as the IC_50_. The results (Fig. 2) showed that the IC_50_ of *N. kaouthia* was 0.0281 µg/ml.

To study the specificity of the nAChR binding, *N. kaouthia* cytotoxin I at various concentrations (0.4875 to 0.0152 µg/ml) was tested as described above. It was shown (Fig. 2) that the cytotoxin had no effect on the nAChR binding. Thus, the inhibition reaction was specific to the postsynaptic neurotoxins.

#### Neutralization of *N. kaouthia* venom by horse monospecific antisera as determined by nAChR binding to the NK3-coated plate

**Figure 3:**
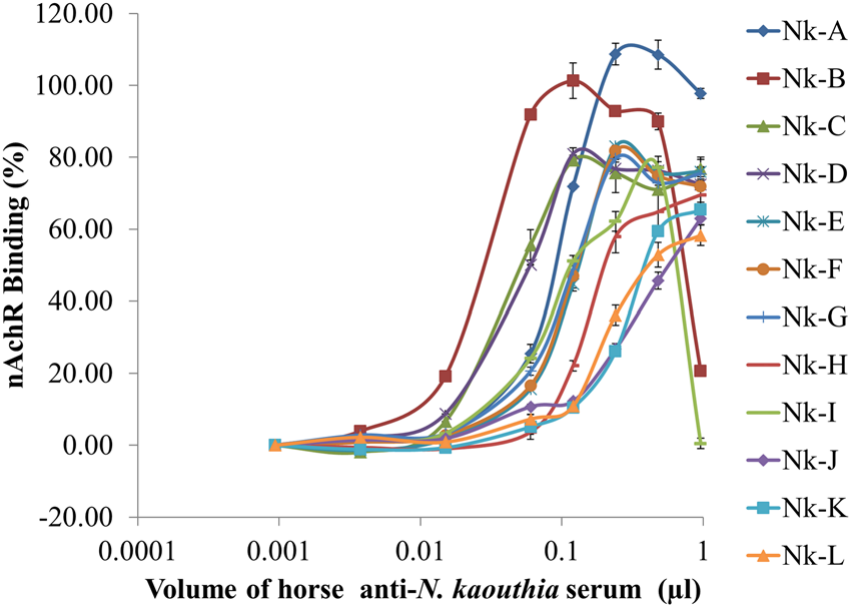
Effects of horse anti-*N. kaouthia* sera in neutralizing *N. kaouthia* venom as determined by nAChR binding to NK3-coated plate. nAChR binding were expressed as mean ± S.D. of 4 determinations.

Twelve horse monovalent anti-*N. kaouthia* sera were serially diluted 2-fold from 1:500 to 1:512,000 and the dilutions were separately incubated with 5xIC_50_ of *N kaouthia* venom (1.4029 µg/ml) in the ‘Pre-incubation 1’ experiment. After ultrafiltration and ‘Pre-incubation 2’, the reaction mixtures were added to the NK3-coated plates. The binding of the free nAChR to the plate was measured at OD_450nm_ and the results are shown in Fig. 3.

**Figure 4:**
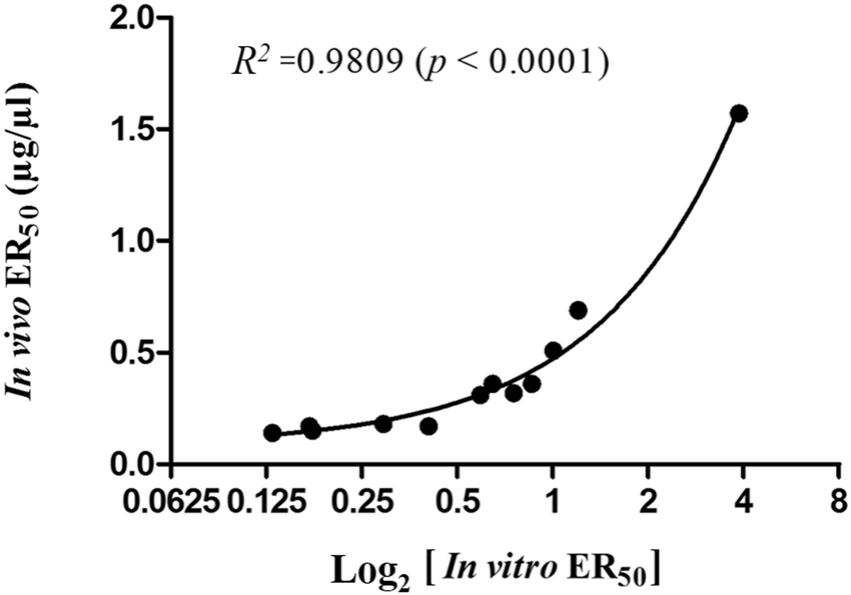
Regression between the nicotinic binding efficacy (Log_2_ [*in vitro* ER_50_]) and the lethality neutralization efficacy (*in vivo* ER_50_). *R* ^2^: Coefficient of determination. *In vitro* ER_50_ values were expressed as mean ± S.D. (μg venom/μl antiserum) of 4 determinations. *In vivo* ER_50_ values were expressed as median dose ± 95% C.I. from serial dose-response study in mice (n = 4–5 mice per dose). Footnote: For each batch of antiserum, the *in vitro* ER_50_ was mean ± S.D. from 4 determinations while the *in vivo* ER_50_ was median ± 95% C.I. (C.I. = confidence level).

The *in vitro* median effective ratio (ER_50_s), expressed as µg of venom neutralized per µl of antiserum, and the *in vivo* ER_50_s (mg of  venom neutralized per ml of antiserum) of the 12 horse antisera are shown in Table 1. The correlation coefficient, *R*, between the *in vitro* ER_50_s and the *in vivo* ER_50_s was 0.9904, and the coefficient of determination for the regression model was *R* ^2^ = 0.9809 (*p* < 0.0001), as shown in Fig. 4.

**Table 1:** *In vitro* and *in vivo* ER_50_s of horse anti-*N. kaouthia* sera in neutralizing *N. kaouthia* venom.

Horse	*In vitro* ER_50_ ± S.D. (µg venom/µl antiserum)	*In vivo* ER_50_ (mg venom/ml antiserum)
Nk-A	0.8598 ± 0.1089	0.36 (0.24–0.54)
Nk-B	3.8693 ± 0.2887	1.57 (1.05–2.36)
Nk-C	1.2039 ± 0.2074	0.69 (0.46–1.03)
Nk-D	1.0055 ± 0.1368	0.51 (0.34–0.76)
Nk-E	0.7526 ± 0.2438	0.32 (0.21–0.48)
Nk-F	0.5911 ± 0.0433	0.31 (0.21–0.47)
Nk-G	0.6456 ± 0.0727	0.36 (0.24–0.53)
Nk-H	0.2921 ± 0.0564	0.18 (0.12–0.27)
Nk-I	0.4060 ± 0.1322	0.17 (0.11–0.26)
Nk-J	0.1307 ± 0.0467	0.14 (0.09–0.20)
Nk-K	0.1748 ± 0.0031	0.15 (0.10–0.23)
Nk-L	0.1712 ± 0.0530	0.17 (0.12–0.26)

For each batch of antiserum, the *in vitro* ER_50_ was mean ± S.D. from 4 determinations while the *in vivo* ER_50_ was median ± 95% C.I. (C.I. = confidence level).

## Discussion

It is reported here the first successful development of an *in vitro* potency assay for antiserum against an elapid snake based on nicotinic acetylcholine receptor binding. The reactions employed in the *in vitro* assay closely mimicked those of the *in vivo* toxicological reactions of the elapid postsynaptic neurotoxins. Unlike the ELISA which at times gave poor correlation with *in vivo* assay and has often been criticized in that the antibody binding did not necessarily result in toxin neutralization, the present *in vitro* assay involved the binding and neutralization by the antisera antibodies of the lethal snake toxins thus preventing them from binding to nAchR. The correlation between the *in vivo* assay using mice and the developed *in vitro* assay was very high as supported by the correlation coefficient of *R* = 0.9904.

An *in vitro* assay based on PSNT binding to nAchR was studied by Stiles^25, 26^ and Alape-Giron *et al*.^12, 27^. Using an ELISA format, the bindings of purified nAchR from *T. californica* to the immobilized long and short PSNTs were shown to be specific. Furthermore, these researchers showed that horse antivenom against *M. nigrocinctus nigrocinctus* venom contained antibodies that inhibited the binding of the venom α-neurotoxins to purified nAchR, and reversed the binding of the toxins already complexed with the receptor^12^. The antivenom ED_50_ in neutralizing the lethal effect of the venom was shown not to correlate with the antivenom’s ability to inhibit the nAchR-binding activity (r = 0.34; *p* > 0.05) but correlated well with the inhibition of phospholipase A_2_ activity. From these results, they concluded that the lethality of the venom was the result of the combined actions of various toxins^12^ and recent proteomics results have shown that the short-chain α-neurotoxins are likely to play a leading role in the lethality induced by this venom^28^.

The *in vitro* assay reported here involved reaction schemes that were different from those reported by Alape-Giron *et al*.^12^. Two salient features of the present assay protocol were as follows.

First, the two crucial reactions i.e., the antibody-venom toxins reaction, and the toxin-nAChR reaction, were carried out in solution rather than on solid surface. This was to allow for total exposure of the reactants’ surface residues resulting in more complete binding with their counterparts, and also to avoid any possible steric interactions between the two high molecular weight reactants (IgG and nAchR) which are likely to be more pronounced on solid surface.

Second, after the venom-antibody reaction in ‘Pre-incubation 1’, the antibodies, free or toxin bound, were removed from the reaction mixture by ultrafiltration. This was important in that if these antibodies were not removed, any free excess antibody remaining in the ‘Pre-incubation 1’ reaction could react with the immobilized toxins in the final reaction resulting in reduced binding of nAChR (added in the later step) to the immobilized toxins. Furthermore, since the dissociation constant of toxin-antibody complexes are usually in the micromolar range while the toxin-nAchR dissociation constant is closer to nanomolar range^29^, it is conceivable that, without the ultrafiltration to remove the antibodies, the antibody-bound toxin might dissociate and form tighter complex with nAChR. These reactions would shift the equilibrium of the toxin-antibody reaction, and the measured amount of nAChR bound to the immobilized toxin would also be reduced.

The described assay procedure was thought to improve the reactions involved and to eliminate or minimize any inaccuracy of reactant concentrations measured; leading to highly correlated *in vitro* and *in vivo* results.

It should be noted in Fig. 3 that at higher concentrations of the antisera Nk-A Nk-B and Nk-I, the nAChR bindings were decreased. This phenomenon, often observed in immunoassays, is known as the ‘prozone phenomenon’ or ‘hook effect’ where, at excess concentration of antibody, immunochemical reactions e.g., hemagglutination, were inhibited or become less pronounce^30, 31^.

The *in vitro* potency assay described here should be applicable to antivenoms against most, if not all, elapids whose venoms contain mainly or exclusively postsynaptic neurotoxins as major lethal components. However, the usefulness of the assay for some elapids producing other lethal toxins e.g., some *Bungarus* venoms may contain, in addition to PSNTs, presynaptic neurotoxins (β-neurotoxins) which are highly lethal^32, 33^. Thus, this *in vitro* nAchR binding assay which worked well with antisera against the *Naja* venoms might not work as well with some of the *Bungarus* venoms, depending on the abundance of the β-neurotoxins present in the particular venom and their role to the overall neurotoxicity.

Since effective, affordable antivenoms against snake venoms remain unavailable in many parts of the world^2^, studies were being made to produce pan-specific antivenoms that cover multiple snake venoms from wide geographical areas^6^. Such a pan-specific antivenom could be produced in large volume and, due to the economy of scale, could be produced at low cost. However, in the development of such pan-specific antivenoms, a large number of mice would be needed to assay its efficacy against many homologous and heterologous venoms, and over the years, the cumulative number of mice used will be even more perturbing considering the need to repeat the assay from batch to batch of antivenom. With the developed *in vitro* assay described here, the development and production of poly-specific or pan-specific AVs should become easier and simpler. This should eventually result in saving the lives of mice and the victims of snake envenomation.

In conclusion, the assay should reduce the use of mice for potency assays for example, during the immunization program and/or fractionation process of antivenom production. In some cases, it may even replace the *in vivo* assay. The *in vitro* assay is less expensive, less biologically variable and could avoid the ethical and religious issues involved. The *in vitro* assay could facilitate the development and production of new and effective antivenoms, especially the pan-specific antivenoms which usually employ a large number of mice. The availability of new antivenoms combined with the reduction in production cost could, in turn, save the lives of more snakebite victims, which are mostly from the poorer regions of the world^34^.

## Materials and Methods

### Materials

Electroplaque tissue from *Torpedo californica* (Pacific electric ray) was obtained from Dr. Charles Winkler, Aquatic Research Consultants (San Pedro, CA, USA). *Naja kaouthia* (NK, formally known as *Naja naja siamensis*) venom from pool of several adult snakes of Thai origin and horse monovalent antisera against *N. kaouthia* was purchased from Queen Soavabha Memorial Institute (QSMI). Benzoquinonium dibromide was purchased from Santa Cruz (Dallas, TX, USA). Goat against rat IgG conjugated with horse radish peroxidase (HRP) was purchased from Abcam (SF, USA). *N. kaouthia* postsynaptic toxin 3 (NK3, formally known as *N. n. siamensis* toxin 3) was purified as described by Karlsson *et al*.^35^. *N. kaouthia* cytotoxin (CTX-I) was purified as described by Tan *et al*.^36^. N-hydroxysuccinamide-Sepharose (NHS-Sepharose) was from GE Health Care. All other reagents were from Sigma Chemical, St Louis. Missouri, unless stated otherwise.

### Methods

#### Purification of nAChR and production of anti-nAChR antibody in rats

Purification of nAChR from *T. californica* electroplaque was carried out as described by Lindstorm *et al*.^37^. The purified receptor **(**10 µg) in 0.1 ml phosphate buffer saline (PBS) pH 7.4 was emulsified with Complete Freund adjuvant and injected subcutaneously into each of the eleven Wistar rats. The second and third immunizations were carried out using the receptor emulsified in Incomplete Freund adjuvant and alum as the adjuvant, respectively. Blood of each rat was collected from the heart at the end of the experiment.

#### *In vivo* neutralizing activity of horse monospecific antisera against *N. kaouthia* venom

The intravenous median lethal dose, LD_50_, of *N. kaouthia* venom, 0.18 (0.12–0.27) µg/g, was adapted from Tan *et al*.^38^ of the same laboratory using the same batch of venom as with the current work. Neutralization of lethality was conducted as described by Ramos-Cerrillo *et al*.^39^. Briefly, a challenge dose of the venom constituting 5 LD_50_ in 50 μl saline was pre-incubated at 37 °C for 30 min with varying dilutions of the pooled horse sera in normal saline, to give a total volume of 250 μl. The venom-antiserum mixture was subsequently injected into the caudal vein of the mice. The mice were allowed free access to food and water *ad libitum* and the number of survival after 48 h was recorded. The effective dose-50 (ED_50_) was determined as the volume of antiserum that protected 50% of the challenged mice from death using probit analysis. The neutralizing efficacy of the antiserum was also expressed as median effective ratio (ER_50_ ± 95% C.I. where C.I. is confidence interval) in mg venom/ml antiserum that gave 50% survival of the mice tested.

### Development of the *in vitro* neutralization assay using nAChR-PSNT binding

#### Optimal conditions of nAChR, rat anti-nAChR antibody and goat-anti-rat HRP conjugate binding to NK3 coated microtiter plate

This assay was the basic assay format for *in vitro* binding of solubilized, purified nAChR to the elapid PSNTs immobilized on the microtiter plate. Briefly, purified NK3 at various concentrations were coated to the microtiter wells (Polystylene High Binding 3590, Costar). After washing with 0.05% TWEEN 20 in phosphate buffered saline (PBST), the plate was blocked with 200 µl/well of PBST and 1% BSA for 2 hr. The purified nAChR (in PBS containing 0.05% Tween 20 and 0.15 BSA) at various concentrations were added to bind the immobilized NK3 by incubation at 25 °C for 1 hour. After 3 time washings to remove the unbound nAChR, rat anti-nAChR serum at various concentrations was added and incubated at 25 °C for 1 hr; this was followed by addition of goat-anti-rat-HRP conjugate (ab7097, Abcam) and incubated for 1 hr at room temperature. After 4 washes with PBST, 100 µl/well of freshly prepared substrate solution (0.01% w/w 3,3′,5,5′-tetramethyl benzidine and 0.003% hydrogen peroxide in 0.075 M citrate buffer, pH 5.0) was added. The plate was allowed to stand in the dark for 30 min at 25 °C and the reaction was stopped by adding 25 µl of 4 N sulfuric acid. The absorbance of 450 nm was read against blank using an ELISA reader (Multiskan Go, Thermo Scientific). Optimal concentrations of NK3 (used for coating the plate), nAChR, rat anti-nAChR antibody and goat-anti-rat-HRP conjugate were estimated and used in the experiments that followed.

#### Inhibition of nAChR binding to the NK3 coated plate by *N. kaouthia* venom

The ability of an elapid venom (*N. kaouthia)* which contains PSNTs to inhibit the binding of nAChR to NK3 immobilized plate was studied and was expressed as IC_50_ (venom concentration inhibiting 50% of the nAChR binding). In this assay, *N. kaouthia* crude venom at various concentrations was pre-incubated (25°C for 1 hr) with a fixed and optimal concentration of nAChR before the mixture was added to the NK3-coated plate and incubated at 25°C for 1 hour. This was followed by additions of rat anti-nAChR serum at 1:1600 dilution and incubated at 25°C for 1 hr, followed by 1:4500 diluted goat-anti-rat-HRP conjugate (Abcam) and incubated for 60 min at 25 °C. A parallel experiment using purified NK3 as the reference standard in place of the venom was also carried out. The concentration of the tested venom used in the pre-incubation step that inhibited 50% of the nAChR binding to the immobilized NK3 was the median inhibitory concentration (IC_50_) of that venom.

#### Inhibition of the *N. kaouthia* venom PSNTs from binding to nAChR by horse antisera

Using a format similar to that described above, an *in vitro* assay of horse antiserum potency (*in vitro* ED_50_) was carried out. Horse sera at various amounts (0.94 nl–0.96 µl) were pre-incubated at 37 °C for 1.5 hr with a fixed amount (5 × IC_50_) of *N. kaouthia* venom in 137 mM NaCl, 2.68 mM KCl, 8.10 mM Na_2_HPO_4_, 1.47 mM KH_2_PO_4_, 0.05% TWEEN20, 0.1% w/v BSA in a total volume of 480 µl. This was referred to as ‘Pre-incubation 1’. The mixture was then filtered through a 100 kDa MWCO ultrafiltration membrane (Amicon^®^) to remove antibody-toxin complexes, free antibodies and some other high molecular weight horse serum proteins. The filtrates (126 µl) containing the remaining free venom PSNTs were then incubated with an optimal amount of nAChR (14 µl in the same buffer) at 25^o^ C for 1 hr as described above and this was referred to as ‘Pre-incubation 2’. The mixtures containing any remaining free nAchR were then added to the microtiter wells immobilized with NK3, followed by the rat anti-nAChR antibody, goat-anti-rat HRP conjugate, etc. The reaction products were then processed as described above. Wells incubated with a non-immune horse serum in place of antisera were included as background control.

The percentage of nAChR binding was then determined using the following formula:

$$ \% {\rm{nAChR}}\,{\rm{binding}}=\frac{({\rm{OD}}\,{\rm{sample}}-{\rm{OD}}\,{\rm{Ag}}\,{\rm{control}})\times 100}{({\rm{OD}}\,{\rm{\max }}\,-\,{\rm{OD}}\,{\rm{Ag}}\,{\rm{control}})}$$

‘OD max’ represented the binding of nAChR (optimal amount) which was not pre-incubated with the venom or antiserum.

‘OD Ag control’ represented the binding of nAChR after being pre-incubated with 5 folds of IC_50_ of *N kaouthia* venom (and without antiserum in ‘Pre-incubation 1’).

‘OD sample’ represented the binding of nAChR after nAChR (optimal amount) was pre-incubated with filtrate from ‘Pre-incubation 1’ (where 5 folds of IC_50_ of *N. kaouthia* venom was pre-incubated with various amount of antiserum).

From the results, dose–response curves of horse sera volumes vs percents of nAChR binding were constructed. The *in vitro* neutralizing activities (ED_50_s) represented the horse antiserum volumes at which the nAChR binding was inhibited by 50 percent compared to wells incubated with buffer in place of antisera. The *in vitro* median effective ratio, ER_50_, represented µg venom/µl antiserum that the nAChR binding was inhibited by 50% was calculated. The results of the *in vitro* study on nAChR binding for every batch of the horse antisera (Nk-A to Nk-L) were presented as means ± S.D. of 4 determinations.

### Ethics approval

Experiment involving rats was reviewed and approved by the Animal Care and Use Committee of the Faculty of Veterinary Science, Mahidol University, Protocol no. MUVS-2014–29 in accordance with the Guidelines of the National Research Council of Thailand. The protocol of animal study on mice was based on the guidelines given by the Council for International Organizations of Medical Sciences (CIOMS) and approved by the Institutional Animal Care and Use Committee (IACUC) of the University of Malaya (Ethical clearance No. 2014-09-11/PHAR/R/TCH).

### Miscellaneous procedures

Protein concentration was determined by the procedure described by Lowry *et al*.^40^ and by BCA Protein assay Kit (Pierce^TM^) using bovine serum albumin as the standard. IC_50_ and ED_50_ values were determined using GraphPad Prism 5.0 program and BioStat 2009 version 5.8.3.0, respectively. The correlation analysis was evaluated by linear regression using GraphPad Prism 5.0 software. In brief, the correlation coefficient *R* was determined from the linear regression model, and *R* ^2^ (coefficient of determination) is the square of the correlation coefficient. An *R* ^2^ of 0.8–1.0 indicates that the regression line well fits the data in correlation. The statistical significance of the correlation test was set at *p* < 0.05.

## References

[1] Kasturiratne A (2008). The global burden of snakebite: a literature analysis and modelling based on regional estimates of envenoming and deaths. PLOS medicine.

[2] Williams DJ (2011). Ending the drought: new strategies for improving the flow of affordable, effective antivenoms in Asia and Africa. J Proteomics.

[3] WHO. Progress in the characterization of venoms and standardization of antivenoms (World Health Organization, Geneva, 1981). 7245916

[4] WHO. Guidelines for the Production, Control and Regulation of Snake Antivenom Immunoglobulins (World Health Organization, Geneva, 2010). 10.1051/jbio/2009043 20950580

[5] Weisser, K. *et al*. Animal welfare aspects in the quality control of immunobiologicals: a critical evaluation of the animal tests in pharmacopoeial monographs. (FRAME for ECVAM and PEI, 1997).

[6] Ratanabanangkoon K (2016). A Simple and Novel Strategy for the Production of a Pan-specific Antiserum against Elapid Snakes of Asia. PLOS neglected tropical diseases.

[7] Maduwage K, Silva A, O’Leary MA, Hodgson WC, Isbister GK (2016). Efficacy of Indian polyvalent snake antivenoms against Sri Lankan snake venoms: lethality studies or clinically focussed in vitro studies. Sci Rep.

[8] Barfaraz A, Harvey AL (1994). The use of the chick biventer cervicis preparation to assess the protective activity of six international reference antivenoms on the neuromuscular effects of snake venoms in vitro. Toxicon.

[9] Fry BG, Wickramaratna JC, Jones A, Alewood PF, Hodgson WC (2001). Species and regional variations in the effectiveness of antivenom against the in vitro neurotoxicity of death adder (Acanthophis) venoms. Toxicol Appl Pharmacol.

[10] Tan KY, Tan CH, Sim SM, Fung SY, Tan NH (2016). Geographical venom variations of the Southeast Asian monocled cobra (Naja kaouthia): venom-induced neuromuscular depression and antivenom neutralization. Comp Biochem Physiol C Toxicol Pharmacol.

[11] Hodgson WC, Wickramaratna JC (2002). *In vitro* neuromuscular activity of snake venoms. Clin Exp Pharmacol Physiol.

[12] Alape-Giron A, Miranda-Arrieta K, Cortes-Bratti X, Stiles BG, Gutierrez JM (1997). A comparison of in vitro methods for assessing the potency of therapeutic antisera against the venom of the coral snake Micrurus nigrocinctus. Toxicon.

[13] Gutierrez JM, Avila C, Rojas E, Cerdas L (1988). An alternative in vitro method for testing the potency of the polyvalent antivenom produced in Costa Rica. Toxicon.

[14] Barbosa CF, Rodrigues RJ, Olortegui CC, Sanchez EF, Heneine LG (1995). Determination of the neutralizing potency of horse antivenom against bothropic and crotalic venoms by indirect enzyme immunoassay. Braz J Med Biol Res.

[15] Heneine LG, Carvalho AD, Barbosa CF, Aravjo dos Santos MR (1998). Development of an ELISA to assess the potency of horse therapeutic polyvalent antibothropic antivenom. Toxicon.

[16] Maria WS, Cambuy MO, Costa JO, Velarde DT, Chavez-Olortegui C (1998). Neutralizing potency of horse antibothropic antivenom. Correlation between *in vivo* and *in vitro* methods. Toxicon.

[17] Rial A, Morais V, Rossi S, Massaldi H (2006). A new ELISA for determination of potency in snake antivenoms. Toxicon.

[18] Rungsiwongse J, Ratanabanangkoon K (1991). Development of an ELISA to assess the potency of horse therapeutic antivenom against Thai cobra venom. J Immunol Methods.

[19] Theakston RD, Reid HA (1979). Enzyme-linked immunosorbent assay (ELISA) in assessing antivenom potency. Toxicon.

[20] Leong PK, Fung SY, Tan CH, Sim SM, Tan NH (2015). Immunological cross-reactivity and neutralization of the principal toxins of Naja sumatrana and related cobra venoms by a Thai polyvalent antivenom (Neuro Polyvalent Snake Antivenom). Acta Trop.

[21] Ibrahim NM, Farid NM (2009). Comparison between Two In Vitro ELISA-Based Assays in the Determination of Antivenom Potency. Journal of Applied Sciences Research.

[22] Barber CM, Isbister GK, Hodgson WC (2013). Alpha neurotoxins. Toxicon.

[23] Changeux JP (1990). The TiPS lecture. The nicotinic acetylcholine receptor: an allosteric protein prototype of ligand-gated ion channels. Trends Pharmacol Sci.

[24] WHO. *Guideline for the management of snake-bites* (World Health Organization, Geneva, 2016).

[25] Stiles BG (1991). A non-radioactive receptor assay for snake venom postsynaptic neurotoxins. Toxicon.

[26] Stiles BG, Sexton FW, Guest SB, Olson MA, Hack DC (1994). Characterization of monoclonal antibodies against Naja naja oxiana neurotoxin I. Biochem J.

[27] Alape-Giron A (1996). Characterization of multiple nicotinic acetylcholine receptor-binding proteins and phospholipases A2 from the venom of the coral snake Micrurus nigrocinctus. FEBS Lett.

[28] Fernandez J (2011). Venomic and antivenomic analyses of the Central American coral snake, Micrurus nigrocinctus (Elapidae). J Proteome Res.

[29] Klett RP (1973). The acetylcholine receptor. I. Purification and characterization of a macromolecule isolated from Electrophorus electricus. J Biol Chem.

[30] Schiettecatte, J., Anckaert, E. & Smitz, J. in *Advances in Immunoassay Technology* (eds Norman H., L. Chiu, & Theodore K. Christopoulos) (2012).

[31] Gillet P, Mori M, Van Esbroeck M, Van den Ende J, Jacobs J (2009). Assessment of the prozone effect in malaria rapid diagnostic tests. Malar J.

[32] Abe T, Alema S, Miledi R (1977). Isolation and characterization of presynaptically acting neurotoxins from the venom of Bungarus snakes. Eur J Biochem.

[33] Pungercar J, Krizaj I (2007). Understanding the molecular mechanism underlying the presynaptic toxicity of secreted phospholipases A2. Toxicon.

[34] Harrison RA, Hargreaves A, Wagstaff SC, Faragher B, Lalloo DG (2009). Snake envenoming: a disease of poverty. PLoS Negl Trop Dis.

[35] Karlsson E, Arnberg H, Eaker D (1971). Isolation of the principal neurotoxins of two Naja naja subspecies. Eur J Biochem.

[36] Tan KY, Tan CH, Fung SY, Tan NH (2016). Neutralization of the Principal Toxins from the Venoms of Thai Naja kaouthia and Malaysian Hydrophis schistosus: Insights into Toxin-Specific Neutralization by Two Different Antivenoms. Toxins (Basel).

[37] Lindstrom J (1980). Purification of acetylcholine receptors, reconstitution into lipid vesicles, and study of agonist-induced cation channel regulation. J Biol Chem.

[38] Tan KY, Tan CH, Fung SY, Tan NH (2015). Venomics, lethality and neutralization of Naja kaouthia (monocled cobra) venoms from three different geographical regions of Southeast Asia. J Proteomics.

[39] Ramos-Cerrillo B (2008). Characterization of a new polyvalent antivenom (Antivipmyn Africa) against African vipers and elapids. Toxicon.

[40] Lowry OH, Rosebrough NJ, Farr AL, Randall RJ (1951). Protein measurement with the Folin phenol reagent. J Biol Chem.

